# Prediction of Respiratory Decompensation in Patients Receiving Home Mechanical Ventilation: Machine Learning Model Development and Validation Study

**DOI:** 10.2196/78941

**Published:** 2025-12-30

**Authors:** Nerea Berbel Casado, Francesc López Seguí, Natalia Muñoz Moruno, Antoni Rosell, Aïda Muñoz Ferrer, Ignasi Garcia Olive, Marina Galdeano Lozano

**Affiliations:** 1 Chair in ICT and Health, Centre for Health and Social Care Research (CESS), Universitat de Vic - Universitat Central de Catalunya, Carrer Miquel Martí i Pol, 1, Vic, Spain; 2 Centre de Recerca en Economia i Salut, Universitat Pompeu Fabra, Barcelona, Spain; 3 Department of Pulmonology, Clinical Directorate of the Thorax Area, Hospital Universitari Germans Trias i Pujol, Carretera de Canyet, s/n, Badalona, 08916, Spain, 34 934 65 12 00; 4 Institut d'Investigació en Ciències de la Salut Germans Trias i Pujol, Badalona, Spain; 5 Universitat Autònoma de Barcelona, Bellaterra, Spain

**Keywords:** home mechanical ventilation, machine learning, noninvasive ventilation, predictive modeling, random forest classifier, remote patient monitoring, respiratory decompensation, Shapley Additive Explanations analysis, SHAP

## Abstract

**Background:**

Chronic respiratory diseases often require long-term ventilatory support, leading to a growing number of patients treated with home mechanical ventilation (HMV). Despite advancements in telemonitoring with real-time tracking of noninvasive mechanical ventilation enabled by integrated software in HMV devices, early signs of respiratory decompensation may go unnoticed, leading to emergency visits and hospitalizations, which burden both patients and health care systems.

**Objective:**

This study aims to develop and evaluate a machine learning–based model capable of predicting respiratory decompensation events, defined as emergency visits or hospitalizations due to acute deterioration in the patient’s underlying respiratory condition. These events reflect episodes of worsening respiratory status that require urgent medical attention. The model uses data from HMV telemonitoring platforms, with the aim of improving patient outcomes.

**Methods:**

This retrospective study analyzed data from 482 patients on HMV monitored via ResMed, Philips, and Breas platforms, collected between March 2021 and November 2024 at the Germans Trias i Pujol Hospital in Catalonia, Spain. Data included device use, compliance, mask leakage, and ventilator settings. Decompensation was defined as emergency department visits or hospitalizations. A windowing strategy captured the 5 weeks prior to events. Multiple machine learning models were trained using grid search to identify the optimal hyperparameters, prioritizing recall in order to minimize false negatives. Models were evaluated using 10-fold cross-validation. Finally, Shapley Additive Explanations (SHAP) analysis was used for model interpretability.

**Results:**

The final dataset included 157 data windows, balanced for positive and negative cases. Among the models tested, logistic regression achieved the highest recall (mean 0.94, SD 0.06, 95% CI 0.90‐0.98) though with moderate accuracy (mean 0.60, SD 0.05, 95% CI 0.56‐0.64). The random forest classifier achieved the best balance across metrics (accuracy: mean 0.66, SD 0.10, 95% CI 0.59‐0.73; recall: mean 0.78, SD 0.15, 95% CI 0.67‐0.89; *F*_1_-score: mean 0.70, SD 0.10, 95% CI 0.63‐0.77). SHAP analysis revealed that higher use, leakage, and compliance in the week before a decompensation event were key predictors, suggesting compensatory behavior or early clinical deterioration. Overall performance remained moderate, reflecting limitations in sample size, incomplete longitudinal records for daily data, and the absence of key physiological measurements.

**Conclusions:**

This study demonstrates the feasibility of predicting respiratory decompensation using data from HMV telemonitoring systems. Tree-based ensemble models, particularly random forests, provided the most balanced performance, while SHAP analysis offered clinically relevant insights. Although performance was moderate, the findings support further development of predictive tools to enable timely telemedical interventions. Limitations include sample size, missing physiological parameters, and a single-center design. Future research should expand to multicenter datasets and incorporate additional clinical variables to enhance model robustness and generalizability.

## Introduction

The increasing life expectancy of the population has led to the chronicization of numerous diseases, including respiratory conditions. As a result, changes in the treatment and monitoring of these pathologies are necessary. Over the years, there has been a significant rise in the number of patients with respiratory diseases that ultimately develop into chronic respiratory failure, requiring oxygen therapy with or without ventilatory support [1,2].

In response to this growing challenge, noninvasive mechanical ventilation (NIMV) has become widely used since the 1980s, prompting the creation of intermediate respiratory care units (IRCUs). In 2005, the Spanish Society of Pulmonology and Thoracic Surgery defined IRCUs as specialized units dedicated to the monitoring and treatment of patients with acute or exacerbated respiratory failure, aiming to provide appropriate respiratory monitoring and treatment through NIMV [3]. Many patients admitted to IRCUs are advised to continue treatment at home with home mechanical ventilation (HMV) devices. Recent technological advancements have enabled the integration of software into HMV devices, facilitating close and continuous patient monitoring. However, despite these improvements, issues such as poor adherence, limited family support, and undetected patient-ventilator asynchronies continue to compromise treatment effectiveness and patient health [4,5].

Previous research suggests that combining telemonitoring and teleintervention could improve health outcomes, with one study reporting a 70% reduction in the risk of cardiovascular events [6], highlighting the broad potential of such integrated approaches. Additionally, changes detected by the integrated monitoring systems in HMV devices may correlate with clinical deterioration in these patients [7,8]. This convergence of HMV data and proven teleintervention strategies highlights the potential of using HMV parameters in the context of telemedicine for early detection of respiratory decompensation, enabling timely adjustment of ventilator settings or remote intervention. Previous studies have shown the potential of machine learning in predicting ventilator-associated complications among critically ill intensive care unit patients, reaching an average accuracy of 0.69 (SD 0.10) [9]. However, this study focuses on invasive mechanical ventilation, incorporating parameters that are not available in HMV applications.

Building on this evidence, this paper aims to assess the feasibility of using machine learning to detect early signs of respiratory decompensation, based on data extracted from HMV software. Respiratory decompensation in this context is defined as emergency department visits or hospitalizations due to acute deterioration in the patient’s underlying respiratory condition, reflecting episodes of worsening respiratory status that require urgent medical attention; therefore, early identification of potential decompensation can significantly reduce preventable emergency visits due to worsening clinical status or quality of life, which also impose a considerable travel burden on frail individuals. The ultimate goal of this approach is to anticipate respiratory decompensation in individuals using HMV by identifying early warning patterns in ventilator data. Future data-driven predictive models integration into telemonitoring workflows could enable timely adjustments of ventilatory parameters or clinical management before an acute event occurs, helping to maintain patient stability, reduce emergency visits and hospitalizations, improve quality of life, and optimize health care resource use.

## Methods

### Patient Recruitment and Inclusion Criteria

This study was conducted at Germans Trias i Pujol Hospital (HUGTiP), a reference hospital in Catalonia (Spain) that serves a population of 1,448,812 people across 70 municipalities. The study included data collected between March 2021 and November 2024. Patients were recruited voluntarily before their inclusion in the study. The study population comprised hospitalized patients who required HMV initiation after an acute exacerbation in the IRCU or in general hospitalization wards at HUGTiP. It also included patients already on HMV who required therapy adjustments before discharge. Additionally, patients attending outpatient clinics who required new HMV initiation or adjustments in follow-up consultations were included in the analysis.

The study population, comprising both women and men, had a mean age of 69.3 years, with most individuals aged between 60 and 80 years. Chronic obstructive pulmonary disease accounted for more than half of the patients treated with NIMV, representing the main indication for ventilatory support. Other underlying conditions included obesity hypoventilation syndrome (often associated with obstructive sleep apnea) and restrictive thoracic or neuromuscular disorders, such as postsurgical thoracic sequelae, amyotrophic lateral sclerosis, and muscular dystrophies.

To ensure appropriate follow-up and reliable data acquisition, only patients residing within the hospital’s coverage area were considered eligible for HMV. Furthermore, patients needed to have a designated caregiver to assist in therapy adherence and monitoring.

### Data Acquisition and Telemonitoring

Patient monitoring was conducted through validated commercial telemonitoring platforms commonly used in HMV: ResMed, Philips, and Breas. These platforms allow for real-time tracking of NIMV therapy. The home respiratory therapy provider, Esteve Teijin, which is responsible for the geographic area of HUGTiP, granted access to its telemonitoring system through a licensed workstation located at the sleep unit in the Pulmonology Department at HUGTiP.

The telemonitoring system facilitated automated data extraction from ventilators, providing detailed information on therapy adherence and device performance through weekly and daily reports. The weekly reports included information on ventilator use patterns, ventilation settings, and device and mask characteristics. More specifically, the extracted data comprised weekly continuous compliance, last recorded values of expiratory and inspiratory positive airway pressure, ventilation mode, ramp activation and duration, device type, mask type, and size. Daily reports focused on key physiological indicators, such as use (in min), apnea-hypopnea index, and leakage levels.

To provide a comprehensive view of patient status, ventilator-derived parameters were supplemented with hospital admission and emergency department visit dates. Consequently, the study included 482 patients for whom both telemonitoring reports and corresponding date records were available.

### Ethical Considerations

This study complies with the approval of the Ethics Committee of HUGTiP (approval code PI-21‐236) and adheres to the ethical principles outlined in the Declaration of Helsinki [10], ensuring the highest standards of research ethics. Informed consent was obtained from all participants (or their legal guardians, where applicable) prior to enrollment. No financial compensation was provided for participation, and involvement was entirely voluntary. To ensure confidentiality, all personal identifiers, including names and medical record numbers, were replaced by the hospital’s information system professionals with unique study codes. These codes were used for data analysis, ensuring that no real patient information is disclosed. Patient autonomy and rights were respected throughout the study, with participant well-being prioritized above scientific or societal interests.

### Data Preprocessing

#### Overview

To capture temporal trends associated with decompensation events, a data preprocessing pipeline was implemented based on the consecutive reports obtained from the telemonitoring system. Both the constant parameters, expiratory positive airway pressure and inspiratory positive airway pressure, and the dynamic variables from the reports that reflect treatment adherence and patient status were selected, which included continuous compliance in the current period, use, apnea-hypopnea index, and leakage. The key steps of the pipeline are described in the following sections.

#### Windowing Strategy

Ensuring that each patient had consecutive reports with complete data was critical to capture the patterns leading up to a decompensation. However, strict continuity requirements risked significant data loss. To overcome this limitation, “windows” were defined spanning from 35 days before a decompensation event up to 7 days before the event date. In addition, for the daily variables, the mean of the available values for the weekdays was calculated. This allowed for the inclusion of patients even if they had some missing values. In patients with multiple decompensation events separated by at least 35 days, each event was treated as an independent window.

#### Reference Group Selection

The reference group was comprised of patients without any emergency or hospital admission, as well as patients with decompensation events, from whom report data were selected only if both the start and end of the window were at least 35 days away from any decompensation event, even when multiple events occurred. This approach ensured that the models would learn temporal patterns predictive of imminent decompensation, rather than simply distinguishing between inherently frail patients—who tend to have more frequent health care encounters and poorer baseline health—and less frail patients. Frail patients often exhibit distinct patterns in ventilator use, such as higher total use, which could confound the model if not properly controlled. By controlling for these differences, the model focuses on true predictors of imminent decompensation rather than patterns related solely to baseline frailty.

### Vector Preparation

Each variable was first rescaled using min-max normalization, computing each feature’s global minimum and maximum across all reports. Next, to emphasize how recent each measurement was relative to a decompensation event, each report was multiplied by a decreasing time-decay weight. The closest report to the event received a weight of 1, while older reports were down-weighted by a constant decreasing factor, up to 0.5 for the oldest report. Finally, the dataset was balanced to ensure representation of both classes.

### Model Development and Evaluation

Different machine learning models were trained with grid search for hyperparameter tuning, ensuring that the best possible parameter configurations were identified. The models included logistic regression (LR), random forest classifier (RFC), decision tree classifier (DTC), naïve Bayes (NB), k-nearest neighbors (KNN), and extra trees classifier (ETC). For each model, a predefined set of hyperparameters was exhaustively explored. For LR, the hyperparameters included the regularization strength *C*, ranging from 0.01 to 100, the penalty types *l1* and *l2*, the solvers *lbfgs*, *liblinear*, and *saga*, and maximum iterations ranging from 1000 to 1,000,000. The RFC and ETC were both tuned by varying the number of estimators between 10 and 500, adjusting the maximum depth from shallow to unrestricted (up to 30), and modifying the minimum number of samples required to split a node or form a leaf, while also exploring different splitting criteria such as *gini*, *entropy*, or *log_loss*. DTCs were similarly optimized by adjusting depth, split, and leaf requirements and splitting criteria to balance model complexity and generalization. KNN was tuned by varying the number of neighbors from 1 to 11, the weighting method between uniform and distance, and the algorithm among *auto*, *ball_tree*, *kd_tree*, and *brute*. Finally, for NB, the variance smoothing parameter ranged from 1·$e^{-12}$ to 1·$e^{-7}$.

Given the clinical context, where minimizing false negatives is crucial, grid search was configured to prioritize maximizing recall, thus ensuring that the number of false negatives is minimized, as failing to identify a positive case is more critical than a false positive in this setting. All models were implemented using the open-source Python (version 3.11.12; Python Software Foundation) library *scikit-learn* [11].

Additionally, two separate neural network models were developed. The first was a neural network (NN) with a depth ranging from 2 to 5 layers, selected based on performance. This model consisted of alternating fully connected layers and rectified linear unit activations, ending with a sigmoid output layer for binary classification. The second model was a recurrent neural network (RNN), composed of stacked RNN layers with tanh activation, followed by fully connected layers and rectified linear unit activations, also terminating in a sigmoid output. The optimal architecture for each model was empirically determined. Both models were trained using binary cross-entropy loss, and the limited-memory Broyden-Fletcher-Goldfarb-Shanno and Adam optimizers were evaluated and compared. The entire pipeline was implemented using the open-source Python library *PyTorch* [12].

Models were evaluated through 10-fold cross-validation, using accuracy, precision, recall, and *F*_1_-score as performance metrics. Results are reported as mean (SD) with 95% CIs.

### Model Interpretation

In this study, the Shapley Additive Explanations (SHAP) method [13] was used to assess the interpretability of the predictive models developed. Once the optimal model was identified, a SHAP explainer was created. This explainer is designed to compute SHAP values, where each value indicates the contribution of a corresponding feature toward the final prediction, thus breaking down the prediction into interpretable parts. Summary plots that display the global importance of features across the entire dataset were generated. These plots rank features by their average impact on predictions and reveal the direction of their influence, making it easier to interpret how changes in feature values affect the model’s outcomes.

## Results

### Windows

The dataset resulted in a balanced sample size of 157 windows; thus, each fold holds 16 windows for testing and the remaining 141 for training.

### Evaluation Outcomes

Results show that LR with *C*=0.1, a maximum of 1000 iterations and the *liblinear* solver, achieved the highest mean recall of 0.94 (SD 0.06; 95% CI 0.90‐0.98) but exhibited a mean overall accuracy of 0.60 (SD 0.05, 95% CI 0.56‐0.64), a mean *F*_1_-score of 0.71 (SD 0.03, 95% CI 0.69‐0.73), and a mean precision of 0.58 (SD 0.04, 95% CI 0.55‐0.61; Table 1). RFC, with 200 trees, a maximum depth of 5, and default splitting and leaf parameters, reached the highest mean accuracy of 0.66 (SD 0.10, 95% CI 0.59‐0.73), along with a mean recall of 0.78 (SD 0.15, 95% CI 0.67‐0.89), a mean precision of 0.66 (SD 0.13, 95% CI 0.57‐0.75), and mean *F*_1_-score of 0.70 (SD 0.10, 95% CI 0.63‐0.77). The ETC, a related ensemble approach, using 50 trees with similar depth constraints, achieved a mean accuracy of 0.62 (SD 0.08, 95% CI 0.56‐0.68), mean recall of 0.81 (SD 0.12, 95% CI 0.72‐0.89), and mean *F*_1_-score of 0.69 (SD 0.07, 95% CI 0.64‐0.74).

**Table 1.** Results of classifier performance across 10-fold cross-validation with hyperparameter settings (N=157).

Model	Hyperparameters	Accuracy, mean (SD, 95% CI)	Recall, mean (SD, 95% CI)	Precision, mean (SD, 95% CI)	*F*_1_-score, mean (SD, 95% CI)
LR^a^	C=0.1 max_iter=1000 solver= ‘liblinear’	0.60 (0.05, 0.56‐0.64)	0.94 (0.06, 0.90‐0.98)	0.58 (0.04, 0.55‐0.61)	0.71 (0.03, 0.69‐0.73)
RFC^b^	max_depth=5 min_samples_leaf=1 min_samples_split=2 n_estimators=200	0.66 (0.10, 0.59‐0.73)	0.78 (0.15, 0.67‐0.89)	0.66 (0.13, 0.57‐0.75)	0.70 (0.10, 0.63‐0.77)
DTC^c^	criterion='entropy' max_depth=None min_samples_leaf=1 min_samples_split=5 min_weight_fraction_leaf= 0	0.57 (0.12, 0.48‐0.66)	0.67 (0.17, 0.55‐0.79)	0.58 (0.09, 0.52‐0.65)	0.61 (0.11, 0.53‐0.69)
ETC^d^	criterion='entropy' max_depth=5 min_samples_leaf=1 min_samples_split=2 n_estimators=50	0.62 (0.08, 0.56‐0.68)	0.81 (0.12, 0.72‐0.89)	0.62 (0.09, 0.56‐0.69)	0.69 (0.07, 0.64‐0.74)
KNN^e^	n_neighbors=3 weights='distance'	0.60 (0.15, 0.49‐0.71)	0.70 (0.20, 0.56‐0.84)	0.61 (0.13, 0.52‐0.70)	0.64 (0.15, 0.53‐0.75)
NB^f^	var_smoothing=1·$e^{-11}$	0.57 (0.09, 0.51‐0.64)	0.62 (0.13, 0.53‐0.71)	0.61 (0.14, 0.51‐0.71)	0.60 (0.08, 0.54‐0.66)
NN^g^	Layers=100,50 solver=‘L-BFGS^i^’	0.61 (0.06, 0.57‐0.65)	0.64 (0.12, 0.55‐0.73)	0.63 (0.07, 0.58‐0.68)	0.63 (0.07, 0.58‐0.68)
RNN^h^	Layers=100,50,16,8,1 solver=‘Adam’	0.61 (0.11, 0.53‐0.69)	0.70 (0.18, 0.57‐0.83)	0.62 (0.12, 0.53‐0.71)	0.65 (0.11, 0.57‐0.73)

a LR: logistic regression.

b RFC: random forest classifier.

c DTC: decision tree classifier.

d ETC: extra trees classifier.

e KNN: K-nearest neighbors.

f NB: naïve Bayes.

g NN: neural network.

h RNN: recurrent neural network.

i L-BFGS: limited-memory Broyden-Fletcher-Goldfarb-Shanno

The single DTC with entropy criterion and unrestricted depth reached a mean accuracy of 0.57 (SD 0.12, 95% CI 0.48‐0.66), mean recall of 0.67 (SD 0.17, 95% CI 0.55‐0.79), mean precision of 0.58 (SD 0.09, 95% CI 0.52‐0.65), and mean *F*_1_-score of 0.61 (SD 0.11, 95% CI 0.53‐0.69). KNN, with three neighbors and distance weighting, produced a mean accuracy of 0.60 (SD 0.15, 95% CI 0.49‐0.71), mean recall of 0.70 (SD 0.20, 95% CI 0.56‐0.84) and mean *F*_1_-score of 0.64 (SD 0.15, 95% CI 0.53‐0.75). NB, tuned with a *var_smoothing* parameter of 1·$e^{-11}$, showed a mean accuracy of 0.57 (SD 0.09, 95% CI 0.51‐0.64) and mean *F*_1_-score of 0.60 (SD 0.08, 95% CI 0.54‐0.66). The neural network with hidden layers of 100 and 50 neurons achieved a mean accuracy of 0.61 (SD 0.06, 95% CI 0.57‐0.65), mean recall of 0.64 (SD 0.12, 95% CI 0.55‐0.73), and mean *F*_1_-score of 0.63 (SD 0.07, 95% CI 0.58‐0.68), while the RNN, comprising 2 *simpleRNN* layers of 100 and 50 units followed by dense layers, yielded comparable metrics (accuracy: mean 0.61, SD 0.11, 95% CI 0.53‐0.69; recall: mean 0.70, SD 0.18, 95% CI 0.57‐0.83; and *F*_1_-score: mean 0.65, SD 0.11, 95% CI 0.57‐0.73).

### Feature Importances

**Figure 1.**
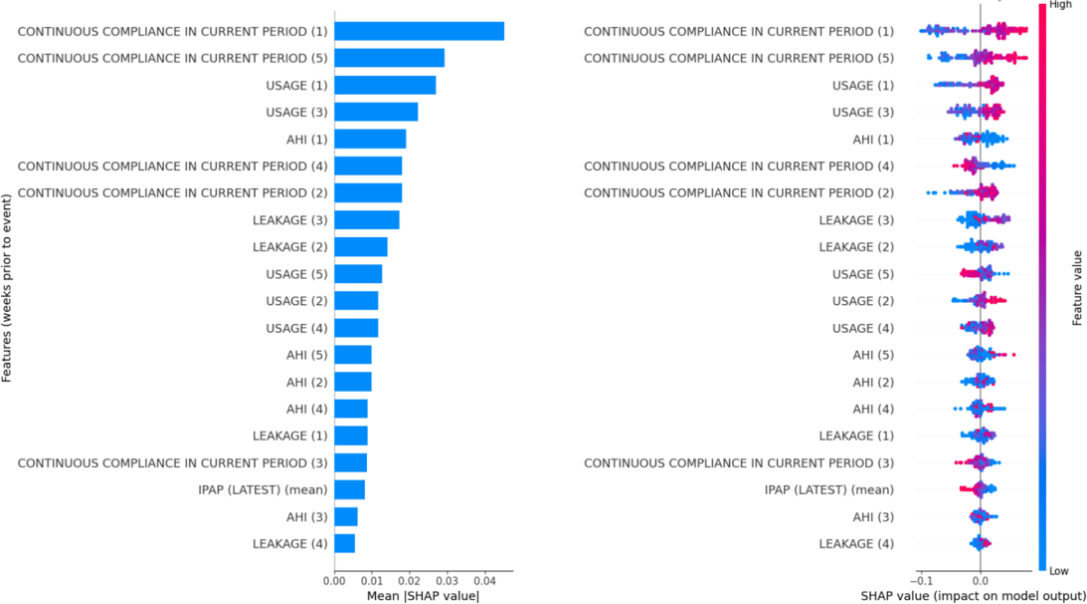
Feature importances and Shapley Additive Explanation (SHAP) values from a random forest classifier (RFC) trained with a fixed train-test split (random seed=42). AHI: apnea-hypopnea index; IPAP: inspiratory positive airway pressure.

As shown in Figure 1, the SHAP interpretation of the RFC. The labels (1) through (5) correspond to the number of weeks preceding a decompensation event: (1) indicates the week immediately before the event, (2) two weeks prior, and so on, up to (5), which represents 5 weeks before decompensation. The SHAP values highlight the importance of use patterns in the model’s predictions. Specifically, higher patient use and continuous compliance during the week immediately prior to the event (1) appear to be strong indicators of respiratory decompensation. Lower compliance in weeks (3) and (4) also contributes to the model’s prediction, while higher compliance in weeks (1), (2), and (5) is similarly associated with increased risk. Elevated leakage values in the period leading up to the event also emerge as relevant features influencing the model’s output.

## Discussion

### Principal Results

Although LR achieved the highest recall (mean 0.94, SD 0.06, 95% CI 0.90‐0.98), it showed lower precision (mean 0.58, SD 0.04, 95% CI 0.55‐0.61) and accuracy (mean 0.60, SD 0.05, 95% CI 0.56‐0.64), indicating a tendency to overpredict positive cases. This may be advantageous in clinical scenarios where identifying all potential positives is prioritized, but it comes at the cost of increased false positives. In contrast, the RFC, configured with 200 trees and a maximum depth of 5, provided a more balanced performance across all metrics, including the highest accuracy (mean 0.66, SD 0.10, 95% CI 0.59‐0.73) and solid values for recall (mean 0.78, SD 0.15, 95% CI 0.67‐0.89), precision (mean 0.66, SD 0.13, 95% CI 0.57‐0.75), and *F*_1_-score (mean 0.70, SD 0.10, 95% CI 0.63‐0.77). This suggests that RFC may be more suitable for general-purpose prediction in this context, balancing sensitivity and specificity.

Other models such as DTC and KNN performed moderately, with KNN exhibiting high variability across folds, indicating sensitivity to the specific train-test splits. DTC and NB recorded the lowest accuracy among all models, while NB also showed the lowest *F*_1_-score. This underperformance of NB suggests that its strong independence assumptions limit its ability to capture data patterns. Ensemble approaches like the ETC also yielded competitive results, reinforcing the robustness of tree-based methods. Both neural networks achieved intermediate performance; however, their capacity to learn more intricate patterns may have been limited by the relatively small sample size. Recurrent models generally require larger datasets and longer temporal sequences to effectively learn temporal dependencies. In this study, the relatively short input sequences and small cohort may have restricted the RNN’s capacity to model temporal dynamics robustly. Larger datasets, finer-grained temporal data, and extended temporal windows could allow the exploration of more advanced sequential architectures, such as attention-based models in future applications.

SHAP analysis from the RFC indicated that increased compliance, use, and leakage in the week preceding decompensation could be meaningful predictors. Although at first glance, it may seem counterintuitive that higher compliance is associated with increased risk, this pattern could reflect increased ventilator dependence in patients whose health is deteriorating. Patients may use the device more intensively as their condition worsens, while elevated leakage could indicate improper mask fitting under higher use. Furthermore, variations in global compliance patterns over time, such as a drop in compliance during periods (3) and (4), may be indicative of behavioral changes that precede decompensation.

While SHAP does not directly assess temporal trends or causality, these insights highlight the potential clinical relevance of use trends, suggesting that both increases and fluctuations in ventilator parameters could serve as early warning signals. Nonetheless, interpretability remains a challenge: models with moderate performance may limit the robustness of conclusions, and potential confounding factors that are not captured in the dataset—particularly the absence of detailed physiological variables—could reduce predictive power or lead to misattributions.

### Limitations

One of the primary limitations of this study is the absence of enough physiological explanatory variables. While many such variables are inherently unavailable from HMV data, respiratory rate, although a common monitored parameter, was not available in the reports used for the analysis. The absence of broader physiological or clinical variables (eg, comorbidities and vital signs) could have limited the models’ ability to account for the full clinical picture, reducing the predictive power.

Another notable constraint is the sample size. Although the dataset comprised 482 patients, and the strategy of constructing windows aimed to maximize the sample size, extracting windows with complete necessary information remained a challenge: generally, complete daily data across all 5 consecutive week reports could not be obtained consistently. For this reason, to address data sparsity, available daily measurements were averaged, as mentioned previously in the methodology. However, this approach could compromise temporal resolution, and due to the characteristics of the missing data, data augmentation techniques were insufficient to overcome this limitation.

Finally, the study is limited by its monocentric design, as all patients were sourced from a single center, which may impact the generalizability of the findings.

### Comparison With Prior Work

Although no studies in the literature directly use HMV parameters to predict respiratory decompensation [6], constructed an RF model that achieved an average accuracy of 0.69 (SD 0.10) for predicting ventilatory complications in critically ill intensive care unit patients, using invasive mechanical ventilation parameters. This performance is comparable to our model, which achieved an average accuracy of 0.66 (SD 0.10, 95% CI 0.59‐0.73) using RFC models.

### Conclusions

This study introduced a novel framework for predicting respiratory decompensation using only HMV data. Ensemble tree models, particularly a random forest, achieved the best balance of metrics (accuracy: mean 0.66, SD 0.10, 95% CI 0.59‐0.73; recall: mean 0.78, SD 0.15, 95% CI 0.67‐0.89), while SHAP analysis revealed that higher use, leakage, and compliance 7 days before, that is, period (1), could be an indicator of respiratory decompensation.

Despite the promising nature of these findings, overall performance remained moderate, reflecting the limitations imposed by incomplete longitudinal daily records and the lack of key physiological measurements. Combined with the relatively small sample size, these constraints position the present results as exploratory rather than ready for clinical application. Nevertheless, the findings suggest the potential of using NIMV data for early decompensation identification. Future research should focus on incorporating more clinical and contextual features and validating larger, multicenter cohorts to improve predictive power. With such advancements, this approach could translate into a clinically useful, noninvasive decision support tool for the proactive management of patients on home ventilation.

## Abbreviations

NB: naïve Bayes
ETC: extra trees classifier
DTC: decision tree classifier
HMV: home mechanical ventilation
HUGTiP: Germans Trias i Pujol Hospital
IRCU: intermediate respiratory care unit
KNN: k-nearest neighbor
LR: logistic regression
NIMV: noninvasive mechanical ventilation
NN: neural network
RFC: random forest classifier
RNN: recurrent neural network
SHAP: Shapley Additive Explanations
